# Imaging Cognitive Impairment and Impulse Control Disorders in Parkinson's Disease

**DOI:** 10.3389/fneur.2021.733570

**Published:** 2021-11-05

**Authors:** Antonio Martín-Bastida, Manuel Delgado-Alvarado, Irene Navalpotro-Gómez, María Cruz Rodríguez-Oroz

**Affiliations:** ^1^Department of Neurology, Clínica Universidad de Navarra, Pamplona, Spain; ^2^CIMA, Center of Applied Medical Research, Universidad de Navarra, Neurosciences Program, Pamplona, Spain; ^3^Neurology Service, Sierrallana Hospital-IDIVAL, University of Cantabria, Satander, Spain; ^4^Cognitive Impairment and Movement Disorders Unit, Neurology Department, Hospital del Mar, Barcelona, Spain; ^5^Clinical and Biological Research in Neurodegenerative Diseases, Integrative Pharmacology and Systems Neurosciences Research Group, Neurosciences Research Program, Hospital del Mar Research Institute (IMIM), Barcelona, Spain; ^6^Barcelonabeta Brain Research Center, Pasqual Maragall Foundation, Barcelona, Spain; ^7^IdiSNA, Instituto de Investigación Sanitaria de Navarra, Pamplona, Spain

**Keywords:** impulse control disorders (ICD), Parkinson's disease dementia (PDD), mild cognitive impairment (MCI), magnetic resonance imaging (MRI), positron emission tomography (PET), single photon computed tomography (SPECT)

## Abstract

Dementia and mild forms of cognitive impairment as well as neuropsychiatric symptoms (i. e., impulse control disorders) are frequent and disabling non-motor symptoms of Parkinson's disease (PD). The identification of changes in neuroimaging studies for the early diagnosis and monitoring of the cognitive and neuropsychiatric symptoms associated with Parkinson's disease, as well as their pathophysiological understanding, are critical for the development of an optimal therapeutic approach. In the current literature review, we present an update on the latest structural and functional neuroimaging findings, including high magnetic field resonance and radionuclide imaging, assessing cognitive dysfunction and impulse control disorders in PD.

## Introduction

Parkinson's disease (PD) is the second most common neurodegenerative disease in the world. Formerly considered to predominately be a movement disorder caused by the degeneration of dopaminergic neurons of the substantia nigra pars compacta (SNc) (1, 2). PD is now accepted to also present with non-motor features as part of the clinical manifestations. Among them, cognitive decline and neuropsychiatric alterations are highly debilitating and frequent.

In fact, the risk of developing dementia is about six times higher in PD patients than in age-and-gender matched populations (3). Importantly, within the first 10 years of PD progression, dementia appears in more than 50% of patients (4), reaching up to 80% in the long-term (3, 5). Furthermore, mild cognitive impairment is highly prevalent in PD (PD-MCI) (mean 26.7%; range 18.9–38.2%) (6–9) and is a risk factor for the development of dementia (PDD) (10). Longitudinal studies have revealed that the conversion to dementia occurs in roughly 25–50% (6, 11) of PD-MCI patients within 5 years.

The pathological basis of PDD is multifactorial, as demonstrated in post-mortem and clinical studies (12). For example, studies have reported dopaminergic neurodegeneration within the medial areas of the SNc, ventral tegmentum areas, and fronto-limbic areas (13), and neurotransmitter dysfunction in the cholinergic projections from the nucleus basalis of Meinert as well as in the serotoninergic and noradrenergic efferent fibres from the raphe nucleus and locus coeruleus, which play an important role in cognitive dysfunction (14–16). Furthermore, alpha-synuclein deposition in the form of Lewy bodies and Lewy neuritis spreading to the amigdalar complex, hippocampus, fusiform gyrus and temporal cortex along with synergistic Alzheimer's disease (AD) pathology with beta-amyloid plaques and phosphorylated tau (neurofibrillary tangles) (17) are also critical in the pathogenesis of the cognitive decline.

Apart from cognitive impairment, psychiatric conditions are also common in PD, affecting the majority of patients during the course of the illness. The most frequent and problematic are affective disorders (depression and anxiety), psychosis (mainly visual hallucinations), apathy, and impulse control disorders (ICDs) (18). The most common ICDs experienced by PD patients include pathological gambling, binge eating, hypersexuality, and compulsive shopping, as well as other impulsive-compulsive behaviours (ICBs), such as punding, hobbyism or walkabout. To further complicate matters, approximately 14–17% of PD patients treated with dopaminergic replacement therapy, in particular with dopaminergic agonists (DA), may develop ICDs (19) although the cumulative incidence can be much higher (over 46%) (20) ICDs often result in devastating financial, legal, or psychosocial problems (19). Unfortunately the management of these behaviours, which typically involves reducing DA treatment, can be challenging and often carries the risk of motor worsening or the development of DA withdrawal syndrome (21).

The development of neuroimaging techniques, including high field structural and functional magnetic resonance (MRI) and nuclear imaging, using positron emission tomography (PET) and single photon emission computed tomography (SPECT), helps in the diagnosis and monitoring of the motor and cognitive impairments associated with PD. Furthermore, neuroimaging can be used to shed light on the underlying pathophysiological aspects of cognitive impairment and neuropsychiatric manifestations, which in turn are associated with high levels of patient disability and morbidity.

In the current review, and for the sake of brevity, we will focus on cognitive decline and ICDs in patients with PD. To this end, we conducted a literature review of existing functional and structural imaging studies in cognitive dysfunction and ICD in PD. We performed a thorough search of the PubMed database selecting for English language articles containing “Parkinson's disease dementia,” “mild cognitive impairment,” “impulse control disorders,” “imaging,” “PET,” and “MRI” published up until the 15th of March, 2021. The abstracts were screened for relevance, and carefully read if they were suitable. This review highlights the imaging modalities that detect consistent brain changes associated with cognitive impairment and ICD in PD.

## Cognitive Impairment and Dementia

### Magnetic Resonance Imaging

#### Grey Matter

Grey matter (GM) abnormalities have been the focus of numerous MRI studies in PD. Methodological approaches have substantially changed over the years. Analytical tools did only allow for regions of interest (ROIs) approach in early studies, which consisted of delineating certain brain areas, measuring their volume, and comparing them among different groups. This approach has been clearly overtaken by whole-brain approaches, which are able to disclose differences in GM volume without an *a priori* hypothesis. There are two main techniques, voxel-based morphometry (VBM) and surface-based analyses (SBA), both of them widely used in current studies. Whereas VBM measures GM volume, SBA is able to measure cortical thickness. As compared to VBM, cortical thickness is more sensitive to cortex changes, possibly because it is less dependent on cortical folding and the overall brain size (22). In the present review, we only include VBM and SBA studies (see Table 1).

**Table 1:** Magnetic resonance imaging studies of cognitive impairment or dementia in Parkinson's disease.

**References**	**Population**	**Radioligand and technique**	**State**	**Main results/findings**
**Grey matter imaging**				
Camicioli et al. (23)	PD-MCI PD-NC	VBM	Resting	↓ hippocampus (AD < PD-NC < PDD < HC) ↓ left hippocampus correlated with recognition memory and MMSE
Brück et al. (24)	PD-NC	VBM	Resting	↓ hippocampus and prefrontal (PD < HC) ↓ left hippocampus correlated with verbal memory ↓ prefrontal atrophy correlated with sustained attention tests
Song et al. (25)	PD-MCI PDD PD-NC	VBM	Resting	↓ bilateral temporal, left prefrontal, insular, right occipital (PDD < PD-MCI < HC) ↓ right parietal, middle frontal, insular, striatum (PDD < PD-MCI < PD-NC) ↓ PCC correlated with disease duration in PDD
Choi et al. (26)	PDD PD-MCI PD-NC	VBM	Resting	↓ substantia innominata (PDD > PD-MCI and PD-NC) ↓ substantia innominata correlates with MMSE, attention and object naming domains.
Beyer et al. (27)	PD-MCI PD-NC	VBM	Resting	↓ hippocampal volume (CA1, CA3 and subiculum area) (PD-MCI, PD-NC < HC) ↓ hippocampal volume correlated with CVLT-2 delayed free recall ↓ right hippocampal CA1 and subicular region correlated with CVLT-2 recognition score
Pagonabarraga et al. (28)	PDD PD-NC	SBA	Resting	↓ parietal, temporal, occipital areas (PDD < PD-MCI < PD-NC) ↓ temporal correlated with attentional and language deficits ↓ occipital correlated with attentional, memory and language deficits.
Lee et al. (29)	PDD PD-MCI PD-NC	VBM	Resting (Longitudinal)	↓ left prefrontal, left insular and CN (PDD converters < PD-MCI no converters) PDD converters associated with ↓ executive function, verbal memory, visual recognition memory
Filoteo et al. (30)	PD-NC	VBM	Resting	↓ medial temporal and frontostriatal areas correlated with memory deficits ↓ frontostriatal volumes correlated with executive function ↓ frontal and occipital volumes with visuospatial function
Kandiah et al. (31)	PD-NC	VBM	Resting (longitudinal)	↓ Hippocampal volume is a risk factor for PD-MCI and PDD
Wen et al. (32)	PD-NC	VBM	Resting (longitudinal)	↓ frontal areas in PDD converters ↓ frontal and parietal areas associated with global cognitive scrores
Foo et al. (33)	PD-NC PD-MCI	VBM	Resting (longitudinal)	↓ right hippocampus at baseline (PD-MCI < HC) ↓ baseline right CA1 correlated with attention ↓ CA 2-3 at follow-up correlated with episodic memory in PDD converters
Low et al. (34)	PD-NC	SBA	Resting (longitudinal)	↓ global hippocampal at baseline predicted PDD ↓ subiculum and fimbria volume correlated with attention and executive functions
Zheng et al. (35)	PD-MCI PDD PD-NC	VBM	Resting (meta-analysis)	↓ left anterior insula in PD-MCI < PD-NC (predictor)
Gasca-Salas et al. (36)	PD-NC PD-MCI	SBA	Resting (meta-analysis)	↑ thinning in bilateral frontal, insular and left middle temporal areas (PD-MCI converters > PD-MCI non-converters > controls)
Chung et al. (37)	PD-MCI PDD PD-NC	SBA	Resting	↑ thinning from posterior cortical area to frontal cortex (PDD converters > PDD non-converters) ↑ thinning in right medial superior frontal and olfactory cortices distinguishes PDD converters from PDD non-converters.
Xu et al. (38)	PD-NC	VBM	Resting (longitudinal)	↓ bilateral hippocampal at baseline (PD-NC < HC) correlated with MMSE ↓ bilateral CA4, ML, GC-DG subfields, and left CA2/3 and right presubiculum subfields at follow-up (PD-MCI < PD-NC) correlated with MMSE and MOCA.
Zhou et al. (39)	PD-MCI PDD	VBM	Resting (longitudinal)	↓right temporal at baseline and left temporal and frontal lobe at follow-up (PDD converters < non-converters)
Donzuso et al. (40)	PD-MCI PD-NC	VBM	Regional	↓ frontal gyrus, precuneus, angular gyrus, temporal lobe and cerebellum (PD-MCI < HC) ↓ frontal gyrus correlated with RCPM ↓ precuneus correlated with accuracy of Barrage ↓ Inferior frontal gyrus with Stroop test.
**White matter imaging**				
Kamagata et al. (41)	PDD PD-NC	DTI	Resting	↓ FA in prefrontal white matter and genu of corpus callosum (PDD < PD-NC) ↓ FA in prefrontal white matter and genu of corpus correlated with MMSE
Hattori et al. (42)	PDD DLB PD-NC	DTI	Resting	↓ FA in bilateral in SLF; ILF, IFO, UNF, CIN, INC, CCA, CRA (DLB, PDD, PD-MCI < PD-NC and controls) ↓ FA in parietal WM areas with MMSE
Deng et al. (43)	PDD PD-MCI PD-NC	DTI	Resting	↓ FA left frontal and right temporal WM (PDD, PD-MCI < PD-NC) ↓ FA left AC and CC splenium correlated with disease duration
Meltzer et al. (44)	PDD PD-MCI PD-NC	DTI	Resting	↓ FA and ↑ MD in SLF, IFU, UNF and corpus callosum (PDD, PD-NC < HC) ↓ FA in anterior WM tracts correlated with executive function ↑ MD in anterior WM tracts correlated with global cognition deficits
Agosta et al. (45)	PD-MCI PD-NC	DTI	Resting	↓ FA in SFO, IFO UNF, genu and body of CC (PD-MCI < HC)
Auning et al. (46)	PD-MCI PD-NC AD	DTI	Resting	↓ FA in WM of temporal-parietal tracts (PD-MCI < HC). No differences PD-MCI vs AD. ↓ FA in WM prefrontal tracts with executive and visuospatial deficits.
Chen et al. (47)	PDD PD-NC	DTI	Resting	↓ FA left hippocampus (PDD < PD-HC) ↑ MD in SLF, SFO, UNF, genu of corpus callosum (PDD > PD-HC) ↓ FA in SLF, SFO and hippocampus correlated with MOCA
Bledsoe et al. (48)	PDD PD-NC	DTI	Resting	↑ MD and AD in anterior segments in CC (PDD > PD-NC) ↑ MD and AD in anterior CC associated with global and specific cognitive domains in PDD.
Chondrogiorgi et al. (49)	PDD PD-NC	DTI	Resting	↓ FA body corpus callosum, cingulum, corona radiate (PDD < PD-NC) ↓ FA and ↑ MD in limbic, prefrontal and CC tracts associated with PD-CRS
Beyer et al. (50)	PDD PD-NC	WMH	Resting	↑ WMH in deep WM and periventricular areas (PDD > PD-NC)WMH is associated with MMSE
Lee et al. (51)	PDD PD-NC AD	WMC	Resting	↑ WMH in PDD > PD-NCWMH correlated with UDPRS, MMSE and PD-CDR
Joki et al. (52)	PDD DLB PD-NC AD	WMC	Resting	↑ WMH in DLB and AD > PDD > PD-HC and HC
Huang et al. (53)	PD-MCI PD-NC	WM	Resting	WHM burden associated with PD-MCI (*p* < 0.05) besides the presence of CV risk factorsPeriventricular WMH burden associated with executive function and visuospatial function
**Functional MRI**				
Lewis et al. (54)	PD-NC PD-MCI	fMRI	Working memory task	↓activity of caudate nuclei during retrieval and manipulation. (PD-MCI < PD-NC)Underactivation of dorsolateral and ventrolateral prefrontal cortex and right putamen
Monchi et al. (55)	PD-MCI	fMRI	Card-sorting task	↓activity in tasks involving the caudate nucleus in DLPFC and VLPFC in PD ↑ activity in tasks that do not require the caudate nucleus in DLPFC and VLPFC, premotor, posterior prefrontal
Seibert et al. (56)	PDCN PDD	fMRI	Resting	No differences in FC of the DMN (PD-NC = PDD)
Baggio et al. (57)	PDCN PD-MCI	fMRI	Resting	↓ connectivity in long-range connections and increased local interconnectedness (PD-MCI < PD-NC)
Lebedev et al. (58)	PD	fMRI	Resting	Executive impairment associated with altered balance between cortical and subcortical processing at rest
Amboni et al. (59)	PD-MCI PD-CN	fMRI	Resting	↓ FC of bilateral prefrontal cortex within left F-P network (PD-MCI < PD-NC). Positive correlation between visuospatial function Z score and left prefrontal cortex ICA z score.
Baggio et al. (60)	PD-MCI PDCN	fMRI	Resting	↓ FC of the DAN with widespread, right sided, frontal/insular areas, thalami and left striatum (PD-MCI < HC). ↓ FC less extensive, and regions of DAN itself and of the right FPN. (PD-MCI < PD-CN)
Gorges et al. (61)	PDCN PD-MCI	fMRI	Resting	↓ intrinsic FC within the DMN, the motor network, and the DAN (PD-MCI < HC) ↓ intrinsic FC preferentially in the DMN, but also in the motor, DAN, VAN, and basal ganglia-thalamic intrinsic functional networks (PD-MCI < PD-CN)
Shin et al. (62)	PDCN PD-MCI (early/late)	fMRI	fResting	↓ FC in parahippocampal gyrus, DLPC temporal, and precuneus; ↑ FC in the inferior frontal, primary motor, and occipital areas (PD-MCI early vs PD-CN) ↓ in the medial frontal areas andcingulate cortex and ↑ FC in the parietal and occipital areas (PD-MCI (longer) vs PD-CN)
Chen et al. (63)	PDCN PD-MCI	fMRI	Resting	↓ in PCC-prefrontal cortex, left parieto-occipital juntion, and right temporal gyrus (PD-MCI < PD-CN) ↓ PCC- left inferior temporal gyrus, hippocampus, inferior parietal lobules and PCC/precuneus (PD-MCI <. HC)
Bezdicek et al. (64)	PD-CN PD-MCI	fMRI	Resting	↓ FC in bilateral superior parietal lobule and precuneus (PD-MCI < PDCN) ↓ interconnectedness of the lentiform nuclei and midcingulate cortex, precuneus, the superior parietal cortex and extended portions of the temporoparietal associative cortex bilaterally (PD-MCI < HC)
Diez-Cirada et al. (65)	PDCN PD-MCI HC	fMRI	Resting	No differences (PD-MCI vs. PDCN) ↓ internetwork FC between the somatomotor and cognitive control networks, somatomotor and visual networks, somatomotor and auditory networks, cognitive control and visual and subcortical and DMN (PD-MCI < HC).
Hou et al. (66)	PD-MCI PD-NC	fMRI	Resting	↓ activity betweem DMN and prefrontal cortex (PD-MCI < PD-CN)
Wolters et al. (67)	PDD PD-MCI PD-NC	fMRI	Resting (meta-analysis)	↓ activity in Dorsomedial prefrontal cortex (within the DMN) in PD-MCI
Fathy et al. (68)	PD-MCI PD-NC	fMRI	Resting	↓ FC of DMN and dorsal anterior insula associated with cognitive performance in PD. ↓ connectivity between dAI and ACC was associated with reduced CAMCOG scales
Pan et al. (69)	PD-MCI PD-NC	fMRI	Resting	↑ FC between dAI and superior parietal gyrus (PD-MCI vs PD-NC) correlated with memory and executive tests ↑ FC between dAI and cingulated gyrus (PD-MCI vs HC) correlated with attention/working memory, visuospatial function, and language

*PD-NC, PD with normal cognition; PD-MCI, PD with cognitive impairment; PDD, PD with dementia; LBD, Lewy Body Dementia; AD, Alzheimer's disease; MMSE, Mini metal State Examination; MOCA, Montreal Cognitive Examination Test; RCPM, Raven Coloured Progressive VBM, Voxel-Based Morphometry; SBA, Surface-based approach; CVLT II, California Verbal Learning Test II; FA, fractional anisotropy; MD, mean diffusivity; AD, axial diffusivity; OCC, Occipital; SLF, Superior longitudinal fasciculus; IFL, Inferior longitudinal fasciculus; SFO, Superior frontooccipital fasciculus; IFO, Inferior frontooccipital fasciculus; UNF, Uncinate fasciculus; CIN, Cingulum; INC, Internal capsule; CCA, Corpus callosum; CRA, Corona radiata.; WMH, White matter connectivity; FC, functional connectivitiy; rs-fMRI, resting state functional magnetic resonance imaging; DMN, default mode network; DAN, dorsal attention network; VAN, ventral attention network,; DLPFC, dorsolateral prefrontal cortex; VLPDF, ventrolateral prefrontal cortex; CAMCOG, Cambridge Cognition Examination*.

Early whole-brain studies found higher levels of atrophy in PDD and PD-MCI patients compared to their cognitively normal counterparts (PD-NC) and control subjects [for review see (70, 71)], particularly in the parietal, occipital, mesial temporal, and frontal lobes, as well as in the hippocampus, amygdala, caudate, putamen, thalamus and substantia innominata. Furthermore, compared to PD-MCI patients, PDD patients exhibit GM reductions in the temporal and prefrontal areas (25), the amygdala (26), the anterior cingulate, the entorhinal and orbitofrontal cortices as well as in the parahippocampus, temporal pole, precuneus, and fusiform and lingual areas (28). A recent meta-analysis of voxel based morphometry (VBM) studies found that PD-MCI patients exhibited greater atrophy in the left anterior insula compared to PD-NC patients (35). However, PD-MCI is a heterogeneous clinical entity in which one or several cognitive domains may be affected. Therefore, the focus of the field over the last few years has been to elucidate what type of PD-MCI confers a higher risk of progression to dementia. Most studies in PD-MCI patients, who were prospectively followed and classified according to conversion (or not) to PDD, found that frontal atrophy was associated with conversion to dementia (36, 72, 73). In fact, PD-MCI patients who converted to dementia in <3 (36) or 4 years (37) had greater widespread atrophy and cortical thinning in the frontal, insular, and left middle temporal lobes at baseline than non-converters, with frontal lobe atrophy being the strongest predictor of progression to dementia (37). These findings were reinforced by a cross-sectional study showing that patients who developed PD-MCI within 2 years of diagnosis exhibited greater atrophy in the superior frontal gyrus than those with later cognitive decline (40). In addition, longitudinal studies in PD-NC patients who converted to PD-MCI patients over time showed greater GM atrophy in the frontal, parietal, and temporal areas (32), as well as in the insular cortex and caudate nucleus (29, 32, 39). Overall, the presence of frontal lobe atrophy seems to be a good predictor for cognitive decline in both PD-MCI to PDD and PD-NC to PD-MCI patients, which in turn is associated with lower cognitive scores on frontal/executive, language, and memory domains (7, 72–74).

Alzheimer's disease (AD)-related pathology is also present in cognitively impaired PD patients, specifically of the amnestic type (75). In fact, the presence of AD-related atrophy, such as hippocampal atrophy, has been described in PD patients with cognitive impairment. Previous studies have found increased hippocampal and entorhinal cortex atrophy in PDD patients compared to non-demented patients (70, 71), which was associated with memory impairment (24, 27, 30). In addition, reduced hippocampal volume has been associated with the development of PD-MCI and PDD in longitudinal studies (29, 31, 76). Recent advances in analytical imaging procedures have allowed the analysis of hippocampal subfields volume, indicating that the atrophy of some regions might confer higher risk of dementia (33, 34, 38).

The association of certain gene variants and cognition and their influence on structural changes have been assessed in some studies. Among genes associated with PD, glucocerebrosidase (GBA) mutations confer the highest risk of dementia. PD patients with GBA mutations as compared to those without GBA mutations experienced a more rapid motor and cognitive decline together with a greater, earlier and faster cortical thinning in posterior parieto-occipital regions as well as frontal and orbito-frontal cortices as demonstrated in longitudinal study (77). The catechol O-methyltransferase (COMT) Val158Met polymorphism has also been associated with cognitive decline. It has been recently shown that PD patients harbouring the Val/Val genotype had widespread reduction in GM, including fronto-subcortical and parieto-temporal territories (78). Finally, microtubule-associated protein tau (MAPT) H1/H1 genotype is considered a risk factor for taupathies in addition to cognitive dysfunction in PD. In an interesting study from Sampedro et al. (79) cross-sectional and longitudinal GM reductions in parieto-temporal areas were found in PD patients with homozygous for MAPT H1 compared to PD patients not harbouring this genetic mutation.

In summary, GM atrophy occurs in the early stages of cognitive decline in PD, and steadily increases along with the progression of cognitive deficits, before broadly affecting the cortical and subcortical areas in the dementia stage. Therefore, atrophy in frontal areas and certain hippocampal subfields might lead to the development of dementia in PD and should be considered as a potential biomarker.

#### White Matter

Several studies have shown that fractional anisotropy (FA) is reduced and mean diffusivity (MD) is increased in the main white matter (WM) tracts (the superior and inferior longitudinal, inferior fronto-occipital, cingulate and uncinated fasciculi, and the anterior limb of the internal capsule) of PDD patients compared to PD-NC patients or controls [for review see (70, 71)]. Similarly, PD-MCI patients exhibit less FA than PD-NC patients or controls in the inferior fronto-occipital and uncinate fasciculi corpus callosum as well as the corona radiate (42–45, 80). Interestingly, a previous longitudinal study observed higher widespread MD in patients with PD-MCI than in those with PD-NC at 18 months follow-up (81), which correlated with lower executive and attention cognitive scores. It has been suggested that WM alterations conveying a cortical-subcortical disconnection may precede GM changes in PD patients during the process of cognitive decline. Indeed, Hattori et al. found prominent WM changes in both PDD and PD-MCI patients, while concurrent GM changes were only observed in subjects with PDD (42).

The role of WM tracts in cognition is further supported by several studies reporting correlations between cognitive functions and WM abnormalities in PD-NC patients. In fact, global cognition has been shown to be correlated with low FA values in the superior and inferior longitudinal fasciculi, the inferior fronto-occipital fasciculus, the corpus callosum, the uncinated fasciculus, and the cingulum (41, 47, 82, 83). Furthermore, impairments in executive function have been consistently found to be associated with WM abnormalities in the frontal and parietal regions (46, 48, 49, 84).

Another, seemingly more imprecise, way of assessing WM integrity is detecting the presence of WM hyperintensities (WMHs), which has yielded heterogeneous results. For example, several longitudinal studies did not find any significant differences in WMHs between PD-MCI, PDD and PD-NC patients (51, 85, 86) or any association between WMHs and clinically relevant cognitive decline in studies (86). However, others studies have reported greater deep WMHs and periventricular WMHs in PDD patients compared to PD-NC patients (50–52) and in PD-MCI to PD-NC patients (68).

In summary, WM integrity is disrupted in the main tracts in PD-MCI and PDD patients. These changes might precede GM atrophy suggesting that abnormalities within key WM tracts may be the first structural changes resulting in functional asynchrony of interconnected brain regions devoted to cognitive function. The value of several WM related metrics, such as FA and MD, in the early diagnosis of cognitive decline deserve further attention.

#### Functional MRI

Functional connectivity (FC) studies assess regional activation of the brain or the level of dependency between two or several anatomic locations through functional MRI (fMRI) in resting state or with the execution of experimental paradigms. The default mode network (DMN) symmetrically involves the medial prefrontal cortex, precuneus, posterior cingulate gyrus, inferior parietal lobes, and lateral temporal cortices (87) and is activated during cognitively demanding tasks requiring higher-order conceptual representations (87). It is the most studied resting-state network in PD, showing enhanced activity during rest and decreased activity during experimental tasks.

Importantly, there is a clear direct association between DMN activity and cognition in PD. For example, a resting-state fMRI study (88) found that PD-NC patients displayed a positive correlation between the connectivity of their right medial temporal lobe and the DMN in the context of memory performance, as well as between the inferior parietal cortex and the DMN in visuospatial performance. A recent meta-analysis found that cognitive impairment in PD was associated with brain FC alterations, predominantly in the DMN (67). Studies in PD-MCI patients have also revealed functional hypoconnectivity of the DMN (61–63, 89, 90), which was positively associated with global cognitive function (63, 68, 91, 92). Interestingly, DMN connectivity with the occipital and posterior parietal cortical regions was found to be increased in PD-MCI patients (60), which in turn was correlated with visuospatial performance and occipital-parietal cortical thinning (57). Findings from other studies suggest that DMN connectivity abnormalities can be used to characterise PD patients, regardless of their cognitive status (66, 93), and that other resting-state networks, such as the fronto-parietal network (FPN), are more specifically linked to PD-MCI (59). Nevertheless, abnormalities in other resting-state networks, such as in the sensorimotor network (SMN) (65, 89), the ventral attention network (VAN) (94), the dorsal attention network (DAN) (64), and the salience network (SN) (69) have been found to be associated with PD-MCI, and with low cognitive performance in PD patients (54, 58, 95). However, differences in pre-processing and analysis methods as well as in the PD-MCI criteria may explain the heterogeneity of these results.

Importantly, fMRI task-based studies have shown that PD-NC patients demonstrate weaker recruitment of several areas, including the anterior cingulate cortex, caudate, putamen, and left precentral gyrus as well as the medial, dorsolateral and ventrolateral prefrontal cortices during working memory or executive function tasks (55, 96, 97). However, those changes are also found in PD-MCI patients, hence they may be associated with the presence of executive dysfunction (54).

Recognition memory is typically impaired in patients with dementia (98). fMRI while performing a verbal memory paradigm PD patients showed a weaker deactivation than controls in the inferior orbitofrontal and temporal cortices that correlated with verbal recognition memory (99).

In conclusion, an altered pattern of resting FC in the DMN seems to be associated with cognitive impairments in PD, which in turn is associated with posterior cortical cognitive deficits that eventually progress to dementia. Functional brain changes might precede structural abnormalities and thus, the value of fMRI in early diagnosis is a promising tool to be considered in further studies.

### Nuclear Imaging

#### Brain Glucose Metabolism

Several [18F] fluoro-D-glucose ([18F]FDG) PET studies with PD-MCI patients show reduced frontal, temporoparietal, occipital and precuneal metabolism as well as the caudate nucleus compared to healthy controls, and in less degree to PD-NC (100–104), being regional hypometabolic changes more marked in multi-domain PD-MCI patients than in single-domain (103). Furthermore, extensive areas of hypometabolism, mostly affecting the posterior cortical regions, including the parieto-occipital, associative parietal, and inferior temporal cortices (102) and to a lesser degree, the striatum and prefrontal cortex (101, 104, 105), have been observed in PDD patients when compared to controls, PD-NC and PD-MCI (see Table 2).

**Table 2:** Radionuclide imaging studies of cognitive impairment or dementia in Parkinson's disease.

**Studies**	**Population**	**Radioligand and technique**	**State**	**Main results/findings**
**Glucose metabolism**				
Huang et al. (106)	PD-MCI PD-NC	PET FDG	Resting	↓ posterior cortical prefrontal and parietal (PD-MCI < PD-NC ↑metabolism in brainstem and cerebellum (PD-MCI > PD-NC)Expression of PDCP (*p* > 0.05)
Hosokai et al. (102)	PD-MCI PD-NC	PET FDG	Resting	↓ posterior cortical regions (temporo-parieto-occipital junction) (PD-MCI < PD-NC, HC)
Pappatá et al. (104)	PD-MCI PD-NC	PET FDG	Resting	↓ prefontal, parietal, associative cortices and striatum (PD-MCI < PD-NC, HC)
Bohnen et al. (100)	PDD PD-NC	PET FDG	Resting (Longitudinal)	↓ caudate, occipital PCC and associative visual cortex (BA 18) in PDD < PD-NC and HC) baseline. ↓ follow up at thalamus, PCC, occipital, parietal and frontal in (PDD < PD-NC).
García-García et al. (86)	PDD PD-MCI PD-NC	PET FDG	Resting	↓ frontal and parietal (PD-MCI < PD-NC); ↓ arietal, temporal and occipital (PDD < PD-MCI) Executive function correlated with parieto-temporo-occipital and frontal metabolism; memory correlated with temporo-parietal metabolism; visuospatial correlated with parieto-temporo-occipital metabolism; Language with frontal metabolism
González-Redondo et al. (105)	PDD PD-MCI PD-NC	PET FDGVBM	Resting	↓ metabolism > atrophy in angular gyrus, occipital, orbital and frontal lobes (PD-MCI > PDD) ↓ metabolism areas replaced by atrophy with widespread hypometabolism (PDD > PD-MCI)
Tard et al. (107)	PDD PD-NC	PET FDG	Resting (Longitudinal)	↓ follow up metabolism bilateral precuneus, left temporal and fusiform gyrus (PDD < PD-NC)
Baba et al. (108)	PDD PD-MCI PD-NC	PET FDG	Resting (Longitudinal)	↓ follow up metabolism bilateral parieto-occipital cortices (PDD < PD-MCI and PD-NC)
**Dopaminergic imaging**				
Rinne et al. (109)	PD-NC	PET[^18^F]fluorodopa	Resting	Put, CN and Frontal cortex (PD < HC) ↓FDOPA in CN correlated with Stroop interference task ↓FDOPA in Frontal cortex correlated with digit span, verbal fluency and recall tests.
Walker et al. (110)	PD-NC DLB AD	SPECT[^123^I]FP-CIT	Resting	Put, CN (PD, DLB < AD, HC)
Ito et al. (111)	PD-NC PDD	PET[^18^F]fluorodopa	Resting	CN, VS and ACC (PDD < PD-NC, HC) ↓DaT in CN correlated with MMSE
Nagano-Saito et al. (112)	PD-NC	PET[^18^F]fluorodopaPET FDG	Resting	RCPM score positively correlated with the FDOPA Ki in the left hippocampus and ACC
Van Beilen et al. (113)	PD-NC	PET[^18^F]fluorodopa	Resting	↓FDOPA in CN correlated with executive, memory and language composite scores
Nobili et al. (114)	PD-NC ET	SPECT[^123^I]FP-CIT	Resting	Caudate and right putamen (PD-NC < ET) ↓DaT in CN correlated with executive score deficits
Polito et al. (115)	PD-NC	SPECT[^123^I]FP-CITPET FDG	Resting	↓DaT in CN correlated with verbal fluency performance ↓DaT in CN modulates hypometabolism in ACC and DLPFC
Niethammer et al. (116)	PD-NC	SPECT[^123^I]FP-CITPET FDG	Resting	Correlation of DAT CN uptake and PDCP expression
Sawamoto et al. (117)	PD-NC	PET[^11^C]-raclopride PET FDG	Resting	↓ RAC binding in ACC and MPFC in PD ↓ dopamine release in CN in PD in working memory task
Christopher et al. (118)	PD-MCI PD-NC	[^11^C]FLB 457 PET[^11^C]-DTBZ PET	Resting	↓ D2 binding in salience network PD-MCI < PD-NC ↓ D2 binding in PHG and insula correlated with memory performance ↓ D2 binding in ACC and insula correlated with executive function
**Cholinergic imaging**				
Bohnen et al. (119)	PDD PD-NC LBD	[^11^C]-PMP AChE	Resting	↓Global cortical AChE of 12.9% in PD-NC, 19.8% in PDD < HC
Hilker et al. (120)	PDD PD-NC	[^11^C]-MP4A AChE	Resting	↓Global cortical AChE of 29.7% in PDD and 10.7% in PD-NC < HC) ↓AChE in left inferior parietal lobule, precentral gyrus, and right PCC (PDD < PD-NC)
Gilman et al. (121)	PD-NC	[^11^C]-PMP AChE	Resting	↓Global cortical AChE of 15.3% (PD-NC < HC) Regional reductions mainly located in temporal, parietal, occipital cingulate cortices as well as amygdala and hippocampus.
Klein et al. (122)	PD-NC PDD LBD	[^11^C]-MP4A AChE^18^F Fdopa^18^FDG PET	Resting	↓Global cortical AChE of 22.6% in PD-NC, 33.2% in PDD < HC Global cortical reductions from frontal to occipital areas PDD < PD-NC and HC
Kotagal et al. (123)	PD-NC PDD LBD	^11^C]-PMP AChE	Resting	↓thalamic AChE of 12.8% in PD-NC, 19.8% < HC
Shimada et al. (124)	LBD AD	[^11^C]-MP4A AChE	Resting	↓Global cortical AChE of 27.8% (LBD < AD) Regional reductions mainly located in temporal, parietal, occipital, cingulate cortices (in order of reduction)
Meyer et al. (125)	PD-NC PD-MCI	[^18^F]-Fluoro-A-85380	Resting	↓Global cortical and subcortical α4β2*-nicotinic acetylcholine receptor PD-MCI < PD, Regional reductions in hippocampus, amygdala, cerebellum, thalamus, and putamen
Colloby et al. (126)	DLB AD	[123I]-5-IA-85380	Resting	↓Global cortical and subcortical nicotinic acetylcholine receptor in left frontal gyri and ACC DLB < AD ↓Global nicotinic acetylcholine receptor correlated with executive tasks.
**Protein deposition**				
**A. Amyloid**				
Edison et al. (127)	PDD LBD PD-NC	[^11^C]PIB-PET	Resting	Amyloid positive were found in PDD (2/12) and DLB (11/13) when compared to PD-NC and healthysRegional amyloid deposition in associative, cingulate cortices and striatum
Jokinen et al. (128)	PDD PD-NC	[^11^C]PIB-PETFDG PET	Resting	No differences in amyloid deposition
Gomperts et al. (129)	PD-MCI PD-NC	[^11^C]PIB-PET	Resting	No differences in amyloid deposition in precuneus at baseline (PD-NC = PD-MCI) ↑PiB retention in precuneus at baseline predicted a greater risk of conversion to PDD.
Petrou et al. (130)	PDD PD-MCI LBD PD-NC	[^11^C]PIB-PET	Resting (meta-analysis)	PiB-positive prevalence: - DLB group: 0.68 (95% CI, 0.55-0.82) - PDD group: 0.34 (95% CI, 0.13-0.56) - PD-MCI and PD-NC groups:0.05 (95% CI, −0.07-0.17)
Shah et al. (131)	PD-NC	[^11^C]PIB-PET	Resting	↑cortical (37%) and striatal (16%)-amyloid deposition (PD > HC)Combined presence of striatal and cortical amyloid associated with lower cognitive z score
Ahktar et al. (132)	PD-MCI PD-NC	[^18^F]-florbetapir	Resting	Amyloid-positive scans do not help for diagnosis of PD-MCI ↑ amyloid in PCC correlated with verbal memory performance ↑ amyloid in precuneus, frontal cortex and ACC correlated with naming perfomace
Fiorenzato et al. (133)	PD-NC	[^18^F]florbetaben	Resting	Amyloid positive 10/48 (21%) in PDRegional amyloid deposition in cortical and subcortical areas associated with reduced MOCA and SDMT
Melzer et al. (134)	PD-MCI PDD PD-NC	[^18^F]florbetaben	Resting	No differences in amyloid deposition (PD-NC = PD-MCI = PDD)Absence of clinical associations
Na et al. (135)	PDD	[^18^F]florbetaben	Resting	Amyloid positive were found in PDD (4/23) ↑ amyloid correlated with executive function
Biundo et al. (136)	PDD PD-MCI LBD PD-NC	[^18^F]flutemetamol	Resting	Amyloid positive was 50% in PD and 50 % in LBD at baseline ↑ amyloid associated with reduced MOCA, MMSE, executive and language scores At follow-up there amyloid was associated with dementia.
**B. Tau**				
Gomperts et al. (137)	PDD LBD PD-NC	[^18^F]T807 PET[^11^C]PIB-PET	Resting	↑ cortical tau in inferior temporal gyrus and precuneus (PDD, LBD > PD-NC) ↑ cortical tau in inferior temporal gyrus correlated with MMSE and CDR
Kantarci et al. (138)	DLB AD	[^18^F]T807 PET[^11^C]PIB-PET	Resting	↑ cortical tau in medial temporal cortex (AD > DLB) ↑ cortical tau in posterior temporoparietal and occipital cortices (DLB > AD)
Buongiorno et al. (139)	PD-NC PDD	[^18^F]-FDDNP	Resting (longitudinal)	↑ cortical tau in lateral temporal cortices in PD-NC with longitudinal progression to PDD
**Neuroinflammation**				
**A. Microglial activation**				
Edison et al. (140)	PDD PD-NC	[^11^C](*R*)PK11195-PET[^11^C]PIB-PETFDG-PET	Resting	↑ microglial activation in ACC, PCC, frontal, temporal, pariental cortices and striatum (PDD > PD-NC and HC)
Fan et al. (141)	PDD AD	[^11^C](*R*)PK11195-PETFDG-PET	Resting	↑ microglial activation correlated with MMSE in AD and PDD
Femminela et al. (142)	PDD AD	[^11^C](*R*)PK11195-PETFDG-PET	Resting	↑ microglial activation in hippocampal/parahippocampal areas were associated with cortical atrophy and metabolism in PDD and ADD.
**B. Astroglial activation**				
Wilson et al. (143)	PD-NC	[^11^C]-BU99008 PET	Resting	↓ astroglial expression in posterior cortical and subcortical areas in PD with moderate-advanced stages Astroglial expression correlated with MOCA in moderate-advanced PD.

*PD-NC: PD with normal cognition; PD-MCI: PD with cognitive impairment; PDD: PD with dementia; LBD: Lewy Body Dementia; AD: Alzheimer's disease; ET: essential tremor; PET: positron emission tomography; SPECT: single-photon emission computed tomography; PDCP: Parkinson's disease cognitive pattern; DAT: dopamine transporter; AAC: anterior cingulate cortex; PCC: posterior cingulate cortex, CN: caudate nucleus; Put; Putamen; VS: ventral striatum; DLPC: dorsolateral prefrontal cortex; MPFC: medial prefrontal cortex; PHG: parahyppocampal gyrus; MMSE: minimental test of Folstein; MOCA: Montreal cognitive assessment test; SMDT; symbol Digit Modalities Test; CDR: clinical dementia rating; RCPM; Raven's coloured progressive matrices*.

Several longitudinal studies have assessed the progression of regional metabolic changes in PD with cognitive dysfunction (100, 107, 108). The aforementioned studies showed severe bilateral hypometabolism in parieto-occipital areas, especially within the visual association cortex (Broadman area 18) and posterior cingulate cortices (100), as well as the fusiform gyrus (107), predicting cognitive decline after more than 2 years follow up, thus heralding the conversion from PD-NC and PD-MCI to PDD.

The role of regional hypometabolism in cognition is further supported by several studies reporting correlations between memory and visuospatial functions in the posterior temporal and parietal regions and also with attentional, executive, and language functions in the frontal regions in patients with PD-MCI and PDD (101, 144). Furthermore, in a prospective study (145) found that the association between reduced regional metabolism in temporoparietal and occipital areas and the presence of visual hallucinations in linked with the conversion from PD-NC to PDD.

Using voxel-based spatial covariance analysis of FDG imaging, previous studies described the presence of a *PD cognition-related pattern* (PDCP) (106) consisting of hypometabolism in the medial prefrontal, premotor, precuneus, and parietal association areas. This pattern increased over time along with cognitive decline (146), and was associated with dopaminergic denervation in the nucleus caudate (116), as well as with executive and memory performance in PD-MCI patients (106). The expression of PDCP has been considered as a potential imaging biomarker for cognitive dysfunction in PD, although its prognostic value is yet to be ascertained (147).

The relationship between cerebral metabolism and atrophy displays dissociable patterns along cognitive impairment in PD, with regional hypometabolism preceding spatially matching structural atrophy areas (105). Thus, in PD-MCI patients, areas with hypometabolism exceed atrophy in the angular gyrus, occipital, orbital, and frontal lobes, however in PPD patients; these hypometabolic areas are replaced by atrophy and widespread cortical and subcortical reductions in metabolism is observed surrounding the atrophy areas. This indicates that there is a specific gradient of severity in cortical changes as cognitive dysfunction progresses in PD, with atrophy lagging behind hypometabolism as the pathological stages continue.

In conclusion, changes in brain glucose metabolism are present at the early stages of cognitive impairment in PD with hypometabolism in posterior parieto-occipital areas in PD-MCI, which steadily extends to the frontal and subcortical areas in PDD. Hypometabolism in the posterior cortex may point to the development of dementia in PD, representing an earlier step to grey matter atrophy.

#### Dopamine

Several studies have suggested that cognitive dysfunction in PD is partially based on striatal dopaminergic degeneration, which leads to dysfunction of the frontostriatal pathways (109, 110, 114, 115). In fact, studies have found that PDD and in less degree PD-MCI patients have a greater striatal dopaminergic deficit than PD-NC patients, as assessed with either ^123^I Ioflupane FP-CIT SPECT (110, 114) or ^18^Flurodopa (F-Dopa) PET (109, 111).

In particular, higher dopaminergic denervation in the caudate nucleus has been found to be associated with dysfunction in working memory, attention, and verbal fluency (109, 112–114) in both PDD and PD-MCI patients. Importantly, the associative fronto-striatal circuitry (orbitofrontal and dorsolateral prefrontal cortices) is known to be modulated by caudate dopaminergic signalling in PD patients suffering from executive dysfunction (115). Dopamine transporter (DAT) binding in the caudate nucleus is associated with the expression of the *PD-related cognitive pattern* (PDCP) (116), highlighting the importance of nigral dopaminergic input in the caudate nucleus and its cognitive functioning in PD. According to previous studies, reduced DAT availability in the caudate nucleus may be used as a potential predictor of cognitive dysfunction in PD (148), but only when combined with other diagnostic biomarkers in a multiple regression analysis including CSF (Aβ_42_ to t-tau ratio) and non-motor clinical scales (Montreal Cognitive Assessment (MoCA) and University of Pennsylvania Smell Identification Test (UPSIT) scores).

Dopaminergic depletion in extrastriatal areas derived from mesocortical and mesolimbic projections is also involved in the cognitive dysfunction associated with PD. For example, previous studies have found that frontal areas, including the anterior cingulate cortex (ACC) and middle frontal gyrus as well as the caudate nucleus, displaying reduced dopaminergic F-dopa-PET uptake in patients with PDD (109, 111, 112) when compared to PD-NC and controls. Moreover, the dopaminergic reduction in frontal areas shows inverse correlation with executive and attentional dysfunction in PDD (109).

Furthermore, the availability of post-synaptic dopaminergic D2 tracers such as ^11^C Raclopride is found to be decreased along the mesolimbic and mesocortical areas in patients with PDD compared to PD-NC and controls (117). In contrast, reduced availability of D2 receptors is showed in bilateral insula, ACC and parahippocampal giry in patients with PD-MCI when compared to PD-NC (118), being in turn associated with executive and memory deficits.

The relationship between striatal dopaminergic degeneration and cortical degeneration is of special interest in PD. In a multimodal study, Sampedro et al. (149) showed that dopaminergic loss in caudate nucleus in early stage PD patients as measured with DAT is associated with reduced cortical thickness in both frontal, temporal and posterior cortices in cross-sectional and longitudinal cohorts, which in turn are associated with neuropsychological deficits. Previous results are important to remark as reduced caudate DAT uptake as well as cortical thickness in temporo-parieto-occipital areas in PD-NC patients could potentially predict the conversion to PD-MCI (70).

In summary, dopaminergic depletion in the caudate nucleus, as well as in the extrastriatal mesocortical and mesolimbic areas, are associated with the progression of cognitive decline in PD. Furthermore, reduced caudate dopaminergic function may be a surrogate marker of cognitive decline in PD, but first and foremost, this deficit indicates the presence of executive dysfunction.

#### Acetylcholine Activity

Cholinergic transmission from the basal forebrain and brainstem (nucleus basalis of Meynert and pedunculo-pontine nucleus, respectively) has been found to be reduced in PD patients (150), suggesting that it plays a relevant role in cognitive dysfunction (150).

Several radioligands that bind the vesicular acetylcholine transporter, analogues of the acetylcholinesterase, and post-synaptic nicotinic and muscarinic receptors have been assessed using SPECT and PET techniques (151).

Patients with PDD and PD-MCI show a significant cortical reduction of cholinesterase activity in the temporal, occipital, parietal, frontal, and anterior cingulate cortices (119, 121, 123, 124), as well as in the amygdala and thalamus (121, 123), compared to PD-NC patients. Interestingly, loss of cortical cholinesterase activity may also occurs in early stages of the disease in *de novo* PD-NC patients showing significant (12%) losses in the medial occipital cortex (121) when compared to healthy controls. In addition, reduction of acetylcholinesterase activity in PDD patients is associated with poorer performance in global cognition (152) as well as working memory and attention deficits (151) but not with the severity of motor symptoms.

Furthermore, significant changes in post-synaptic Ach receptors have been found parallel cognitive dysfunction in PD patients (151). PD-MCI had reduced binding of nicotinic receptors in the thalamus, temporal and parietal cortices as well as hippocampus when compared to PD-NC and healthy controls, which in turn was associated with the severity of the cognitive deficit when measured with global cognitive scales (125). Importantly, post-synaptic cholinergic receptors may display compensatory increase, no change, or a decrease probably due to degeneration of non-cholinergic systems, such as noradrenergic and serotoninergic systems, to which these receptors are coupled (153) which has to be taken into account in the interpretation of the findings.

It is important to note that both dopaminergic and cholinergic dysfunction provide divergent contributions to cognitive dysfunction in PD according the “dual-syndrome” hypothesis (154). In fact, multi-radiotracer studies (120, 122) have showed cholinergic denervation and glucose hypometabolism is present in the neocortex from the frontal to the occipital areas in PDD patients, as well as minimal cholinergic denervation in PD-NC patients, compared to controls. On the other hand, dopaminergic denervation in the striatum, limbic, and associative cortices is found in both PD-NC and PDD. The relationship between cortical cholinergic loss and striatal dopaminergic denervation in PDD suggests that cognitive decline in PD appears when the disease spreads from SNc neurons to the cortex, hence the presence of cholinergic dysfunction facilitates the appearance of cognitive decline in PD.

In conclusion, cholinergic imaging in PD patients suffering from cognitive impairments offers an interesting approach for understanding the pathophysiological aspects of PD, especially when used in combination with dopaminergic and glucose tracers.

#### Protein Deposition Imaging

##### β-Amyloid

The development of β-amyloid specific tracers using ^11^C- Pittsburg compound B (PiB) and other radiotracers (^18^F-Florbetaben and ^18^F-Florbetapir) have provided a means for measuring *in vivo* amyloid pathology. To date, several studies have reported heterogeneous results in PDD and PD-MCI patients, taking into account “amyloid positivity” as AD-range of cortical amyloid deposition with PET imaging using PiB. Some studies observed the complete absence of amyloid (127, 128, 134) while others showed mild to moderate amyloid deposition (130, 131) in PDD and PD-MCI patients compared to controls.

Importantly, due to the small sample size of the studies, a meta-analysis (130) found substantial variability of PiB positive results in PD patients with cognitive impairment, with higher levels of binding in patients with Lewy body dementia than in patients with PDD and PD-MCI compared to controls.

Cross-sectional and longitudinal amyloid PET studies show significant associations between cortical amyloid load and global cognitive decline as well as executive dysfunction in PDD patients (133, 135) with respect to PD-NC and controls. However, other studies have observed an association between memory performance with amyloid load in PDD patients (132). One longitudinal PET study (129) found that baseline amyloid binding predicted the severity of cognitive dysfunction over time in PD-NC. Furthermore, a recent prospective amyloid PET study (136) reported that cortical amyloid deposition in PDD and Lewy body dementia is associated with global cognitive deficit as well as language and attention-executive dysfunction when compared to PD-MCI and PD-NC. Interestingly, Shah et al. showed that the combination of amyloid deposition in the striatum and cortex is associated with greater cognitive impairment than amyloid deposition only in the cortex (131).

In summary, although β-amyloid deposition as measured by PET is not always observed in the brain of PDD patients, its presence may predict the presence of cognitive decline and dementia over time.

##### Tau

The recent development of selective and high affinity radioligands capable of binding to tau, such as ^18^F-AV-1451, has paved the way for the assessment of tau deposition in PD patients with cognitive impairment. A cross-sectional study by Gomperts et al. found that increased ^11^F-AV-1451 binding was present in the precuneus and inferior temporal gyrus only in patients with PDD, which in turn was associated with an impairment in global cognitive scales (137).

To date, a few double tracer studies have examined the co-pathology between amyloid and tau in PD. The presence of tau deposition in the posterior cortical areas is in line with previous studies reporting global β-amyloid deposition in PDD patients, compared to those with PD-NC (138). In addition, a previous study found that tau binding was increased in patients with Aβ-positive scans compared to those with Aβ-negative scans, suggesting that tau and β-amyloid deposition display parallel patterns of deposition. Interestingly, in this study (155) tau deposition did not differ in PD-NC, PD-MCI patients, and normal controls.

A longitudinal PD study (139) using another radiotracer (^18^F-FDDNP) that binds both amyloid and tau, reported increased baseline lateral temporal binding in PD-NC patients who eventually progressed to PDD, suggesting that the basal deposition of tau and amyloid is associated with poorer future cognitive function in PD.

Although, to date, only a few imaging studies have measured tau deposition in PD, their findings suggest that it is increased in PDD patients; whereas, they found tau deposition to be relatively absent in PD-MCI and PD-NC patients. In addition, cortical tau deposition is higher with concomitant β-amyloid deposits, indicating the feasibility of detecting *in vivo* co-pathology of protein deposition as demonstrated in post-mortem studies (17).

#### Neuroinflammation Imaging

Neuroinflammation has been reported to be associated with the loss of dopaminergic neurons in the SNc of PD patients (156). Microglial cells can structurally and functionally change when they are activated by the presence of diverse agents, such as oxidative stress, α-synuclein protein aggregation, and neurodegeneration (157). It is thought that activated microglia may display a dual role, both protective and deleterious, thus enhancing the chronic neuroinflammatory process (156). However, whether the progressive neurodegeneration is associated with increased activation of microgliosis remains unclear (158). Nevertheless, post-mortem studies have observed increased microglial activation in the limbic and cortical regions of PDD patients (159). Thus, *in vivo* measurements of microglial activation have begun to be pursued over the last few years.

Importantly, the expression of mitochondrial translocator protein (TSPO) is known to be associated with microglial activation. In fact, first generation TSPO tracers, such as ^11^C-(R)PK11195, have revealed increased cortical binding in PDD patients predominating in the posterior cortical regions, which is associated with reduced cortical metabolism, as measured with 18F-FDG, and with low global clinical cognitive performance (140, 141). Microglial activation has also been shown to be correlated with cortical atrophy in the hippocampus and parahippocampus in PDD patients (142). Due to the non-specific binding of ^11^C-(R) PK11195, new second-generation TSPO radioligands have been developed, including ^11^C-DPA713 and ^18^F-FEPPA. However, to date, there have been no studies using these second-generation TSPO ligands to assess cognitive decline in PD (160, 161).

Astrocytes are the most abundant glial cells in the brain. Similar to microglia, astrocytes change in function and number in the presence of oxidative stress, neurodegeneration, and other factors (162). However, little is known about the role of astrogliosis and the development of cognitive impairment in PD.

Imaging of glial fibrillary acidic protein (163), an astrocytic intermediate filament, with ^11^C-BU990088 (143) revealed widespread binding in the brainstem and cortex in early PD-NC patients compared to controls. In the same study, patients with moderate-late stage PD were observed to have reduced astrocyte expression in the posterior cortical and subcortical areas. They also found that glial fibrillary acidic protein expression was positively associated with global cognitive scores, suggesting a neuroprotective and compensatory mechanism of astroglial activation.

Due to the small number of microglial imaging studies, as well as the lack of specificity of the radiotracers used, the possible role of microglial activation in the cognitive dysfunction associated with PD remains unknown. Similarly, the involvement of astroglial activation in PD is beginning to emerge (164). The recent development of new TSPO radioligands and astroglial tracers will allow researchers to study the role of glial cells in the cognitive decline associated with PD more effectively.

## Neuroimaging of Impulse Control Disorders in Patients with Parkinson's Disease

### Magnetic Resonance Imaging

#### Grey Matter

Whole brain studies using VBM and SBA have also been undertaken in PD patients suffering from abnormal impulsivity. There is some evidence pointing towards higher cortical thickness in PD patients with ICD (PD-ICD) in the ACC, rostral pole and OFC compared to PD patients without ICD (PD-nonICD) (165–167). However, other studies have shown reduced cortical thickness in PD-ICD patients in the inferior frontal gyrus and pars orbitalis (168, 169) or a lack of corticometric changes between PD patients with or without ICD (170, 171). In a prospective study (172) found a small area of increased atrophy the anterior limb of the left internal capsule adjacent to the left caudate nucleus in PD-ICD when compared to the PD-nonICD, with no other significant cortical changes. Interestingly, Tessitore et al. (167) described positive correlations between cortical thickness in the ACC and OFC and ICD severity scores (see Table 3).

**Table 3:** Magnetic resonance imaging and radionuclide imaging studies of cognitive impairment of impulsive control disorders in Parkinson's disease.

**References**	**Population**		**Radioligand and technique**	**State**	**Main results/findings**
**Magnetic Resonance Imaging**					
**Grey Matter Studies**					
Biundo et al. (168)	PD-ICD		Structural MRI	SBA	↑ cortical thinning in fronto-striatal circuitry and ↑ in the left amygdala (PD-ICD)
Pellicano et al. (166)	PD-ICD		Structural MRI	SBA	↑of cortical thickness in rostral ACC and frontal pole (PD-ICD)
Yoo et al. (173)	PD-punding		Structural MRI	VBM	Atrophy in dlPFC area spreading to OFC (PD-ICD punding)
Tessitore et al. (167)	PD-ICD		Structural MRI	VBM	No findings Thicker cortex in ACC and OFC correlated with ICD severity (PD-ICD)
Tessitore et al. (170)	PD-ICD		Structural MRI	VBM	No differences
Imperiale et al. (169)	PD-ICB		Structural MRI	SBATractography	Left precentral and superior frontal cortical thinning, and motor and extramotor white matter tract damage (PD-ICD)
Hammes et al. (165)	PD		Structural MRI	SBA	CT and severity of PD-ICD were positively correlated in the subgenual rostral ACC
**White Matter Imaging**					
Yoo et al. (73)	PD-ICD		Structural MRI	DTI	↑FA in corpus callosum, internal capsule, PCC and right thalamus (PD-ICD)
Canu et al. (174)	PD-ICD punding		Structural MRI	Tractography	Alteration in left pedunculopontine tract and splenium of the corpus callosum (PD-ICD punding)
Mojtahed Zadeh et al. (175)	PD-ICD No treatment		Structural MRI	Diffusion MRI connectometry	Disrupted connectivity in the network of connections between cerebellum, basal ganglia, cortex, and its spinal projections (PD-ICD)
**Functional MRI**					
**a) Rs-fMRI and FC**					
Carriere et al. (176)	PD-ICD		Structural MRI	FC	Functional disconnection between the left anterior Pur and left inferior temporal gyrus and the left ACC
Tessitore et al. (170)	PD-ICD		Rs-fMRI	FCLongitudinal	↓FC in DMN and central executive network and ↑FC in salience during follow-up (PD-ICD)
Tessitore et al. (177)	PD-ICD		Rs-fMRI	FCTransversal	↑FC in salience and DMN, which correlates with ICD severity (PD-ICD) ↓FC in frontal executive network (PD-ICD)
Ye et al. (171)	PD-ICD		Rs-fMRI	FC + AD administration	In somatosensory network: ↓FC between caudate and other cortical regions
Petersen et al. (178)	PD-ICD/ICB		Rs-fMRI	FC	↑FC between VS and ACC, OFC, insula, putamen, globus pallidum (PD-ICD)
Imperiale et al. (169)	PD-ICD		Rs-fMRI	FC	ICD severity and duration modulate FC between somatosensory, visual and cognitive networks (PD-ICD)
Hammes et al. (165)	PD		Rs-fMRI	FC	PD patients with more severe ICB had a ↓ FC between rostral ACC and the nucleus accumbens
Navalpotro-Gomez et al. (179)	PD-ICD		Rs-fMRI	DNFC	Dynamic functional engagement of local connectivity involving the limbic circuit and increased local efficiency in all the aforementioned areas (ICD+)
Koh et al. (180)	PD-high impulsivity (HI)		Rs-fMRI	FC	↑FC between the right frontoparietal network and medial visual network (PD-HI)
Mata-Marin et al. (181)	PD-HS		Rs-fMRI	FC	↑salience network activity with significant ↑ in the right IFG (HS+). Functional disconnection between associative and limbic striatum with precuneus and superior parietal lobe (HS+)
**a) fMRI during task or stimulus**					
Rao et al. (182)	PD-ICD		Perfusion fMRI	Balloon Analogue Risk Task	↓BOLD activity in the right VS during risk taking and significantly ↓ resting CBF in the right VS (PD-ICD)
Frosini et al. (183)	PD-PG		fMRI with visual reward	Gambling-related visual cues/ neutral stimuli/rest periods	↑activation in bilateral ACC, medial and superior frontal gyri, precuneus, right inferior parietal lobule and VS (PD-ICD)
Voon et al. (184)	PD-ICD (PG or CB)		fMRI with task	Gambling taskDA administration	↑more risky choices in the “Gain” relative to the “Loss” condition along with ↓ OFC and ACC activity (ICD+) ↑ sensitivity to risk along with ↓ VS activity (ICD+)
Politis et al. (185)	PD-HS		fMRI with visual reward	Visual sexual cuesOn/off	↑activation in regions within limbic, paralimbic, temporal, occipital, somatosensory and PFC cortices and correlated with increased sexual desire in VS, ACC and OFC (HS+) Off: ↓activation during stimuli
Petersen et al. (178)	PD-ICD/ICB		fMRI with pharmacologic stimuli and task	AD administrationReward learning	↑FC between amígdala and midbrain ↑FC between VS and ACC, not with punishment-avoidance learning
Girard et al. (186)	PD-HS		fMRI with visual stimuli	Delay-discounting of erotic rewards On/off	↑delayed visual stimuli in *on* PD-HS Association between VS, vmPFC and PCC
Paz-Alonso et al. (187)	PD-ICD		fMRI during task	Iowa Gambling Task	↑activation in subcortical and cortical regions typically associated with reward processing and inhibitory control (PD-ICD) Association between ICD severity and regional activations in the right insula and right IFG, mediated by FC with the right VS (PD-ICD)
Haagensen et al. (188)	PD-ICD		fMRI during task	Seuqential gambling taskOn/off	↓”continue-to-gamble” activity in right IFG and subthalamic nucleus (PD-ICD) Individual risk-attitude scaled positively with “continue-to-gamble” activity in right subthalamic nucleus and striatum (PD-ICD) Dopaminergic therapy ↓ FC between IFG and subthalamic nucleus during “continue-to-gamble” decisions and attenuated striatal responses towards accumulating reward
**Radionuclide imaging**					
**Glucose metabolism**					
Tahmasian et al. (189)	PD-ICD		PET FDG	Resting	Patients with ↑ impulsivity ↑ metabolism in OFC, ACC and right insula
Verger et al. (190)	PD-ICD		PET FDG	Resting	Right middle and inferior temporal gyri (ICD+>ICD) ↑connectivity of these areas with OFC. ↓connectivity with right parahippocampus and with the left caudate (PD-ICD)
Marin-Lahoz et al. (191)	PD-ICD		PET FDG	Resting	↑metabolism in widespread areas comprising PFC, both amygdalae and default mode network hubs (PD-ICD > PD-nonICD-) ↓metabolism in right caudate (PD-ICD < HC)
**Molecular studies focusing dopaminergic system**					
**a) Dopamine transporter (DaT) or F-Dopa**					
Cilia et al. (192)	PD-PG		SPECT [^123^I]FP-CIT	Resting	↓VS (PD-ICD < PD-nonICD)
Joutsa et al. (193)	PD-ICD		PET [^18^F]fluorodopa	Resting	↓Medial OFC (PD-ICD < PD-nonICD) No striatal differences
Voon et al. (194)	PD-ICD		SPECT [^123^I]FP-CIT	Resting	↓VS (PD-ICD < PD-nonICD)
Lee et al. (29)	PD-ICD		PET [^123^I]FP-CIT	Resting	Right vmPFC (PD-ICD < PD-nonICD) Tendency left accumbens nucleus (PD-ICD < PD-nonICD)
Vriend et al. (195)	PD-ICD		SPECT [^123^I]FP-CIT	Resting (longitudinal)RetrospectivePD naïve	↓VS (PD-ICD < PD-nonICD)
Smith et al. (196)	PD-ICD		SPECT [^123^I]β-CIT	Resting (longitudinal)ProspectivePD naïve	↓CN and right Put (PD-ICD < PD-nonICD) ↓Total striatum (PD-ICD < PD-nonICD)
Premi et al. (197)	PD-ICD		SPECT [^123^I]FP-CIT	Resting	↓ Left Put and IFG (PD-ICD < PD-nonICD) Functional desconnection between basal ganglia and contralateral ACC (PD-ICD)
Navalpotro-Gomez et al. (198)	PD-ICD	QUIPQUIP-RS	SPECT [^123^I]FP-CIT PET FDG	Resting	VS (PD-ICD < PD-nonICD) ↓DaT in VS associated to ↓metabolism in PFC and ACC (PD-ICD)
Hammes et al. (165)	PD	QUIP-RSBIS	^18^F-DOPA-PET	Resting	↓ Dopamine synthesis capacity in the nucleus accumbens was associated with ICB
Hinkle et al. (199)	PD-ICD	QUIP	SPECT [^123^I]FP-CIT VMAT2 PET (18F-AV133)	Resting	Right striatal VMAT2 ↑ (PD-ICD) Normalizing VMAT2 with DaT SBR strengthened bidirectional correlations with ICD (high VMAT2/DaT) in all striatal regions bilaterally
**b) Studies with dopaminergic receptors**					
Boileau et al. (200)	PD		PET [^11^C]-raclopride [^11^C]-(+)-PHNO	Resting	VS (PD < HC) GP (PD < HC) Putamen (PD>HC)
Payer et al. (201)	PD-ICD		PET [^11^C]-(+)-PHNO	Resting	VS (ICD+ < ICD-) Dorsal striatum (ICD+>ICD-) Negative correlation between VS with ICD severity (ICD+)
Stark et al. (202)	PD-ICD		PET [^18^F] fallypride	Resting	VS (ICD+ < ICD-) Putamen (ICD+ < ICD-)
**Task related studies**					
**a) Activation studies**					
van Eimeren et al. (203)	PD-PG		PET H_2_(15)O	Before and after 3 mg apomorphineCard selection game with probabilistic feedback	↓activity with DA in left OFC, amygdala and ACC (PG+)
Antonelli et al. (204)	PD		PET H_2_(15)O	Before and after 1 mg PMX Delay discounting task, Go/ No Go Task	DA ↑medial PFC and PCC and ↓ in the VS in cognitive impulsivity tasks
**a) Molecular studies focusing dopaminergic system with task**					
Steeves et al. (205)	PD-PG		PET [^11^C]-raclopride	Gambling task	PD-PG ↑ dopaminergic release in VS during gambling
O'Sullivan et al. (206)	PD-ICD/ICB		PET [^11^C]-raclopride	Reward-related cues and L-dopa challenge	↑ Dopaminergic release in VS in ICD/ICB+ following reward-related cue exposure and L-Dopa challenge
Ray et al. (207)	PD-PG		PET [^18^F] fallypride	Gambling task	↓ Dopamine in ACC during control task, not during gabling task in PD-PG ↑ Dopamine in SN and in the TVA
Wu et al. (208)	PD-ICD single or multiple		PET [^11^C]-raclopride	Reward-related visual cues/neutral visual cues	↑ Dopamine release in VS in both single and multiple ICD patients in response to reward cues

*PD-PG, PD patients with pathological gambling; G-SAS, Gambling Symptom Assessment Scale; BIS, Barratt Impulsiveness Scale 11; MRI, magnetic resonance imaging; VBM, voxel-based morphometry; SBA, Surface-based analysis; OFC, orbitofrontal cortex; PD-ICD, PD patients with impulse control disorder; MIDI, Minnesota impulse disorder inventory; QUIP, Questionnaire for Impulsive-Compulsive Disorders; CT, cortical thickness; ICB, impulse control behaviours; ACC, anterior cingulate cortex; IFG, inferior frontal gyrus; dlPFC, dorsolateral prefrontal cortex; TCI-R, Temperament and Character Inventory-Revised; DTI, diffusion tensor imaging; FA, fractional anisotropy; PRS, Punding Rating Scale; rs-fMRI, resting state-functional MRI; CF, functional connectivity; DMN, default-mode network; DA, dopaminergic agonists; PD-CB, PD patients with compulsive buying; VS, ventral striatum; PD-HS, PD patients with hypersexuality; MGS, Massachusetts Gambling Screen, DDS, dopaminergic dysregulation syndrome; PCC, posterior cingulate cortex; ASBPD, Ardouin Scale of Behaviour in Parkinson's disease; SPECT, single-photon emission computed tomography; PD-ICD, PD patients with impulse control disorder; OFC, orbitofrontal cortex; SOGS, South Oaks Gambling Scale; PFC, prefrontal cortex; ACC, Anterior cingulate cortex; PCC, posterior cingulate cortex; BIS, Barratt Impulsiveness Scale 11; PET, positron emission tomography; MIDI, Minnesota impulse disorder inventory; SRMI, Self-Report Manic Inventory; QUIP, Questionnaire for Impulsive-Compulsive Disorders; VS, ventral striatum; vmPFC, ventromedial prefrontal cortex; GP, Globus pallidus; SAST, Sexual Addiction Screening Test; G-SAS, Gambling Symptom Assessment Scale; DA, dopaminergic agonists; PMX, pramipexol; ICB, impulse control behaviors; DDS, dopaminergic dysregulation syndrome; SN, substantia nigra; VTA, ventral tegmental area*.

In summary, morphometric studies have not yet reached conclusive results in PD-ICD patients although it might be that changes in grey matter volume are associated with lack of inhibition related to ICD behaviours in PD.

#### White Matter

Diffusion tensor imaging (DTI) tractography studies have reported widespread WM tract damage in PD-ICD. In particular, increased radial and axial diffusivity of the genu of corpus callosum, uncinate fasciculus, parahippocampal and pedunculopontine tracts in PD-ICD patients as compared to PD-nonICD and controls, regardless of depression and apathy severity (169, 173–175). However, a recent study found that although PD-ICD patients had increased FA in several WM tracts, the WM regions known to be involved in reward- related behaviours were preserved (173).

In summary, only few DTI studies are available in the literature, thus future diffusional imaging studies are needed in order to ascertain the role of WM integrity in ICD.

#### Functional MRI

Resting fMRI studies in PD-ICD patients have observed reduced or enhanced activation in regions known to support cognitive control and inhibition of inappropriate behaviours, such as the PFC, OFC, inferior frontal cortex and ACC (165, 178, 181, 203, 209). In fact, RS fMRI studies have reported both reduced (165, 171, 176) or increased (178, 180) cortico-striatal FC in areas of the limbic circuit as well as others brain-wide networks including the salience, executive, and default-mode networks (169, 170, 177, 181, 210). Interestingly, these studies support the idea that dopaminergic medication is able to alter limbic cortical signals to the VS, impairing the ability to change behavioural focus in response to a change in stimulus salience (177, 178, 186).

A recent studying using a dynamic functional network connectivity approach found dynamic functional engagement of local connectivity involving the limbic circuit, which led to the inefficient modulation of emotional processing and reward-related decision-making (179). It is worth mentioning that there have been very few studies assessing the topological characteristics of brain networks in these patients using graph theory analysis (171, 190). The studies above suggest that, in PD-ICD patients, connectivity is dysfunctional within and between dopaminergic neuronal circuitries involving disrupted communications between important subcortical and limbic-cognitive cortical regions. This implies that the neural mechanisms associated with ICDs in PD patients span molecular to system levels, which are complex and dynamic, and that they cannot already draw a clear and complete picture of ICDs in PD patients.

Previous fMRI studies using reward-related tasks in PD-ICD patients have reported discrepant results. While two studies pointed towards diminished activation in the right VS, OFC and ACC (182, 184), three other studies reported higher activation in the VS, anterior prefrontal cortex (PFC), ACC, and OFC (183, 185, 186). Interestingly, a recent study proposed a hypothesis for this cortico-subcortical interaction, suggesting that the right VS plays a critical role in modulating the functional dynamics of inhibitory-control in frontal regions when PD-ICD patients face penalties (187) pointing to the possibility that these non-unidirectional changes are mediated by various psychological and neural mechanisms.

Furthermore, previous studies have investigated the role of dopaminergic medications during the execution of an ICD-related task. For example, one study performed in PD-ICD patients with and without dopaminergic medication during a gambling task reported medication-independent and medication-related differences in neural activity, which may set a permissive stage for the emergence of ICD during dopamine replacement therapy in PD patients (188).

In conclusion, altered patterns of resting FC in regions involved in cognitive control and inhibition of inappropriate behaviour are associated with ICD in PD, with an important putative effect of dopaminergic medication in the FC between areas of the limbic system and VS participating in the inhibitory-control in the reward circuitry.

### Nuclear Imaging

#### Glucose Metabolism

Studies with ^18^F-FDG PET in ICD show heterogeneous methodology, which can lead to some discrepant findings. Some of them have evidenced higher metabolic rates in the OFC, AAC, and insula in PD patients with higher impulsivity scores (189), with increased connectivity between the parahippocampus and the caudate (190) in patients with ICD respect to non-ICD. A recent systematic review stated that medicated PD-ICDs show increased metabolism in OFC and cingulate cortices, VS, amygdala, insula, temporal and supramarginal gyri (210). In the same line, a recent study suggests that brain metabolism is more preserved in PD-ICD patients than in patients without ICD, which could be related to ICD development (191). In contrast, a single study in PD-ICDs patients reported an association of lower DAT availability in the VS with lower FDG uptake in several cortical areas belonging to the limbic and associative circuits as well as in other regions involved in reward and inhibition processes (198). All these evidences can be matter of debate regarding metabolic studies in general population, which have largely demonstrated that, the hypometabolism of brain regions from “control networks” such as the PFC or the ACC could increase their vulnerability to relapse since it would interfere with cognitive inhibition.

Taken together, available data suggest that ICDs in PD patients are associated with functional alterations (with the influence of dopaminergic treatment) within the mesocorticolimbic network that could affect the control of impulse and lead to impaired inhibitory mechanisms. Although most studies show hypermetabolism in areas of mesocorticolimbic system involved with inhibition and cognitive control networks, one study looking at the relationship of cerebral metabolism and dopaminergic denervation found hypometabolic limbic and associative areas which in turn correlated with the severity of dopaminergic degeneration in the ventral striatum.

#### Dopamine

The most severe dopaminergic cell loss in PD patients occurs in the ventrolateral SNc, leading to dopaminergic deficits mostly in the posterior putamen, ultimately affecting the function of the motor circuit of the basal ganglia (211). However, molecular neuroimaging studies in PD-ICD patients, have revealed that patients also have decreased dopaminergic innervation in the ventral striatum (VS), as measured by DAT SPECT and PET (165, 192, 196–199, 212, 213). Nevertheless, not all studies reported previous finding (193). Moreover, reduced mesolimbic DAT availability has been reported even before the emergence of ICDs, indicating that it may be a predisposing factor for the development of these disorders (195, 196) once dopaminergic treatment is initiated.

Interpretation of altered DAT binding can sometimes be confusing because of two reasons. First, DAT availability may not correlate with dopaminergic neuron counts in PD patients (214). Second, its variation can reflect a functional downregulation in order to increase available dopamine in the synapse, given that the striatal dopamine synthesis capacity in PD-ICDs patients is not reduced, compared to matched PD-nonICDs (215).

On the other hand, functional molecular studies indicate that PD-ICDs patients have a higher release of dopamine in the VS during reward-related tasks (205, 206, 208). Moreover, there is also some evidence of a negative association between the VS dopamine synthesis capacity and ICD severity. Importantly, dopaminergic changes can be also measured outside the striatum. In fact, extrastriatal D2/D3 dopamine receptors can be measured using high-affinity radiotracers (such as [18F] fallypride or [11C] FLB-457). For example, one study in PD patients with pathological gambling showed a reduction in [11C] FLB-457 binding potential in the midbrain during gambling, where D2/D3 receptors are dominated by autoreceptors, along with low dopaminergic tone in the anterior cingulate cortex (ACC) (216).

Taken together, mounting evidence suggests that abnormal dopaminergic innervation or tone in the VS and possibly in the mesocortical circuit are key factors in the development of ICD in PD patients, and could potentially be used in the future as biomarkers for identifying patients at risk of developing such abnormal behaviour when exposed to dopaminergic agents, especially DA.

## Conclusion

In the current review, we highlighted the available and emerging MRI and radionuclide imaging (PET and SPECT studies) techniques used to assess cognitive impairment and ICD in PD. Although several limitations of the aforementioned studies are worth mentioning, including the literature review is not systematic, sample sizes are limited in some studies and different experimental designs and analysis techniques have been used, their findings still shed light on the potential usefulness of imaging for early diagnosing and monitoring the cognitive and neuropsychiatric symptoms of PD. Nevertheless, multimodal multimodal functional and structural longitudinal studies in early PD patients in large well-defined cohorts using advanced method of analysis are still needed in order to better predict the risk of dementia and ICD in PD patients, better understanding of pathophysiology as well as develop novel therapeutic interventions to improve patient care.

## Data Availability Statement

The original contributions presented in the study are included in the article/supplementary material, further inquiries can be directed to the corresponding author/s.

## Author Contributions

AM-B, MD-A, and IN-G: wrote up the original manuscript. MR-O: conception, supervision of the study, and reviewed the final manuscript. All authors contributed to the article and approved the submitted version.

## Conflict of Interest

AM-B, MD-A, and IN-G declare that the research was conducted in the absence of any commercial or financial relationships that could be construed as a potential conflict of interest. MR-O received financial support for her research from national and local government institutions in Spain (Carlos III Institute of Health, Navarra Government) and honoraria from Insightec, and Boston Scientific for lectures, travel and accommodation to attend scientific meetings.

## Publisher's Note

All claims expressed in this article are solely those of the authors and do not necessarily represent those of their affiliated organizations, or those of the publisher, the editors and the reviewers. Any product that may be evaluated in this article, or claim that may be made by its manufacturer, is not guaranteed or endorsed by the publisher.
